# Metal-Ions Intercalation Mechanism in Layered Anode From First-Principles Calculation

**DOI:** 10.3389/fchem.2021.677620

**Published:** 2021-05-10

**Authors:** Junbo Zhang, Xiaodong Lu, Jingjing Zhang, Han Li, Bowen Huang, Bingbing Chen, Jianqiu Zhou, Suming Jing

**Affiliations:** ^1^Department of Energy Science and Engineering, Nanjing Tech University, Nanjing, China; ^2^Department of Electric Power Engineering, Nanjing Normal University Taizhou College, Taizhou, China; ^3^Department of Mechanical and Power Engineering, Nanjing Tech University, Nanjing, China

**Keywords:** layer structure, first-principles, metal-ions battery, structural evolution, MoS_2_

## Abstract

Layered structure (MoS_2_) has the potential use as an anode in metal-ions (M-ions) batteries. Here, first-principles calculations are used to systematically investigate the diffusion mechanisms and structural changes of MoS_2_ as anode in lithium (Li)-, sodium (Na)-, magnesium (Mg)- and Zinc (Zn)-ions batteries. Li and Na ions are shown to be stored in the MoS_2_ anode material due to the strong adsorption energies (~−2.25 eV), in contrast to a relatively weak adsorption of Mg and Zn ions for the pristine MoS_2_. To rationalize the results, we evaluate the charge transfer from the M-ions to the MoS_2_ anode, and find a significant hybridization between the adsorbed atoms and S atoms in the MoS_2_ anode. Furthermore, the migration energy barriers of M ions are explored using first-principles with the climbing image nudged elastic band (CINEB) method, and the migration energy barrier is in the order of Zn > Mg > Li > Na ions. Our results combined with the electrochemical performance experiments show that Li- and Na-ions batteries have good cycle and rate performance due to low ions migration energy barrier and high storage capability. However, the MoS_2_ anode shows poor electrochemical performance in Zn- and Mg-ions batteries, especially Zn-ion batteries. Further analysis reveals that the MoS_2_ structure undergoes the phase transformation from 2H to 1T during the intercalation of Li and Na ions, leading to strong interaction between M ions and the anode, and thus higher electrochemical performance, which, however, is difficult to occur in Mg- and Zn-ions batteries. This work focuses on the theoretical aspects of M-ions intercalation, and our findings may stimulate the experimental work for the intercalation of multi-ions to maximize the capacity of anode in M-ions batteries.

## Introduction

The rapid development of eco-friendly batteries will bring huge benefits to electrical vehicles and capacity devices (Manoj et al., 2018), and the rechargeable batteries with high energy density and long cycle life have attracted considerable attention in terms of improving the energy storage efficiency. Lithium (Li)-ions batteries represent a highly attractive and challenging alternative to rechargeable batteries. While many important achievements have been achieved for these batteries (Ju et al., 2019; Wang et al., 2020), the content of Li is limited in the Earth's crust, which will affect the extensive applications of Li-ions batteries (Schmuch et al., 2018; Sun et al., 2019), suggesting the necessity of the research of multivalent metal-ions (M-ions) batteries.

M (Li, Na, Mg, and Zn)-ions batteries have been demonstrated as very promising rechargeable batteries (Forsyth et al., 2019), and in order to enhance their capacity, many research efforts have been devoted to designing electrodes and solid electrolytes, including the nanostructured Si, transition metal oxides, and layered structure (Hu et al., 2019, 2020a). However, the ability to maintain high capacity and long cycle remains a bottleneck for M-ions batteries, especially anode materials, with a stable anode as the key component in M-ions batteries (Hu et al., 2020a). Therefore, it should be noted that finding an anode with fast ion and electron conduction is the biggest challenge in improving the performance of M-ions batteries.

Interestingly, due to their large surface-to-volume ratios, two-dimensional materials have currently become the research focus in nanostructured anode materials and have been successfully applied in M-ions batteries based on theoretical and experimental studies (Wang et al., 2018; Mohanapriya and Jha, 2019; Hu et al., 2020b). For example, layer anode materials, such as MoS_2_, WS_2_, borophene and graphene, were investigated for Li-ions batteries, and they exhibited good electrochemical performance as the anodes of Li (Na)-ions batteries (Xie et al., 2015). In particular, MoS_2_ nanoplate anodes in Li-ions batteries have been demonstrated to possess a capacity of 1,062 mAh g^−1^ (Cui et al., 2018). Previous investigations have attributed the high performance of the MoS_2_ anode behavior to its unique structural characteristics, such as the layered structure, which can provide more ions channels and storage compartments (Hu et al., 2014; Wang et al., 2017). However, the electrochemical performance of MoS_2_ is poor in divalent-ions (Mg, Zn) batteries. Thus far, few studies have been performed on the diffusion mechanisms and structural changes of the layer anode in M (Li, Na, Mg and Zn)-ions batteries to describe its different electrochemical behavior.

In this work, the adsorption and diffusion mechanisms of Li, Na, Mg, and Zn ions on the ordinary MoS_2_ (2H) structure were explored based on the density functional theory (DFT) and experimental analysis. In addition, the storage capability of Li, Na, Mg, and Zn ions in the MoS_2_ material were predicted and verified by experiments. Moreover, the structural deformation and electronic properties of MoS_2_ during ions intercalation were also investigated. Our theoretical and experimental results show that when used as the anode material of M-ions (Li and Na) batteries, MoS_2_ had significantly less variation in volume during the cycling process, while in Mg and Zn-ions batteries, MoS_2_ exhibited poor electrochemical performance due to the high migration energy barrier and low adsorption energy caused by changes in the structural properties based on first-principles. Furthermore, the MoS_2_ anode was found to undergo a phase transition from 2H to 1T during the intercalation of Li and Na ions, which was difficult to occur during the intercalation of Mg and Zn ions. This phase transition contributes to improving the performance of the MoS_2_ anode in M-ions batteries. Our results facilitate the understanding of the mechanisms of ions diffusion and structural changes of layer materials and provide useful information for designing high-performance anode materials, especially multivalent M-ions batteries.

## Computational and Experimental Methods

### Computational Methods

First-principles were used to describe the ions behavior in the anode based on density functional theory (DFT) with the Vienna ab initio simulation package (VASP) code (Kresse and Furthmüller, 1996). In addition, Perdewe-Burkee-Ernzerhof (PBE) generalized gradient approximation and the projected augmented wave (PAW) method were used to describe the ion-electron interactions in our systems (Perdew and Yue, 1986; Filippi et al., 1996). In this study, the plane-wave cutoff energy was set to 450 eV, and van der Waals corrections (optPBE-vdW) were adopted during structural optimization for the layer materials, and the vdWs interactions were described exactly by using DFT-D3 correction method of Grimme's scheme (Grimme et al., 2011). During the optimization, the Brillouin zone was represented by Monkhorst-Pack (MP), and the k-point mesh of 8 × 8 × 2 was used. Finally, the ion migration energies were acquired using the climbing-image nudged elastic band (CINEB) method (Henkelman et al., 2000; Yao et al., 2017). In our calculation, the structural optimization was considered complete when the force convergence criterion was <0.03 eV Å^−1^ and the total energy per unit cell was within 10^−5^ eV. CI-NEB calculations were performed with linear interpolating 5 images between the initial and final states of the diffusion paths, and spring constants is set as −5. The geometry and energy of the images were then relaxed until the largest norm of the force orthogonal to the path was <0.03 eV Å^−1^. To estimate the adsorption energy of M-ions on the MoS_2_ anode material, the adsorption energy was calculated by the equation: E_b_ = (E_total_ − E_MoS_2__ − nE_M_)/n, where E_total_ is the ground energy of M-ions adsorbed on MoS_2_; E_MoS_2__, the ground energy of MoS_2_; E_M_, the chemical potential of M (Li, Na, Mg, or Zn) atoms; n, the number of M atoms (Tian et al., 2020).

### Experimental Methods

In a typical synthesis, 1.0 mmol ammonium molybdate [(NH4)6Mo7O24] and 28 mmol thiourea (CH4N2S) were mixed with 60 mL deionized water containing 1.0 g PVP, followed by stirring for 2 h and transferring the solution into a 100 mL Teflon-lined stainless-steel autoclave at 200°C for 24 h. After cooling down to room temperature, the black precipitate was obtained by centrifugation, washed several times with water and ethanol, dried at 80°C overnight, and collected as the MoS_2_ material (Li and Peng, 2018).

For the electrochemical measurement, CR2032 cells were assembled in an argon-filled glove box by mixing the active samples, super-P and polyvinylidene fluoride at a weight ratio of 7: 2: 1. Next, the slurry was coated on Cu foil and dried at 60°C under vacuum for 10 h. After cutting into 12 mm discs, the working electrodes were obtained. Meanwhile, lithium (Li) metal or metal sodium (Na) was used as the anode, and the separators were commercial polypropylene (Celgard 2500 membrane) for Li-ions batteries and glass fiber (Whatman) for Na-ions batteries. For Li-ions batteries, the electrolyte was obtained by dissolving 1 M LiPF6 in ethylene carbonate (EC) and dimethyl carbonate (DMC) with 1:1 vol %. For Na-ions batteries, the electrolyte was prepared by dissolving 1 M NaClO4 and 5% fluoroethylene carbonate (FEC) in EC and DMC with 1:1 vol %. The electrochemical performances were recorded on Land cell test station (CT2001A) within the potential range of 0–3 V (vs Li/Li+).

## Results and Discussions

**Figure 1:**
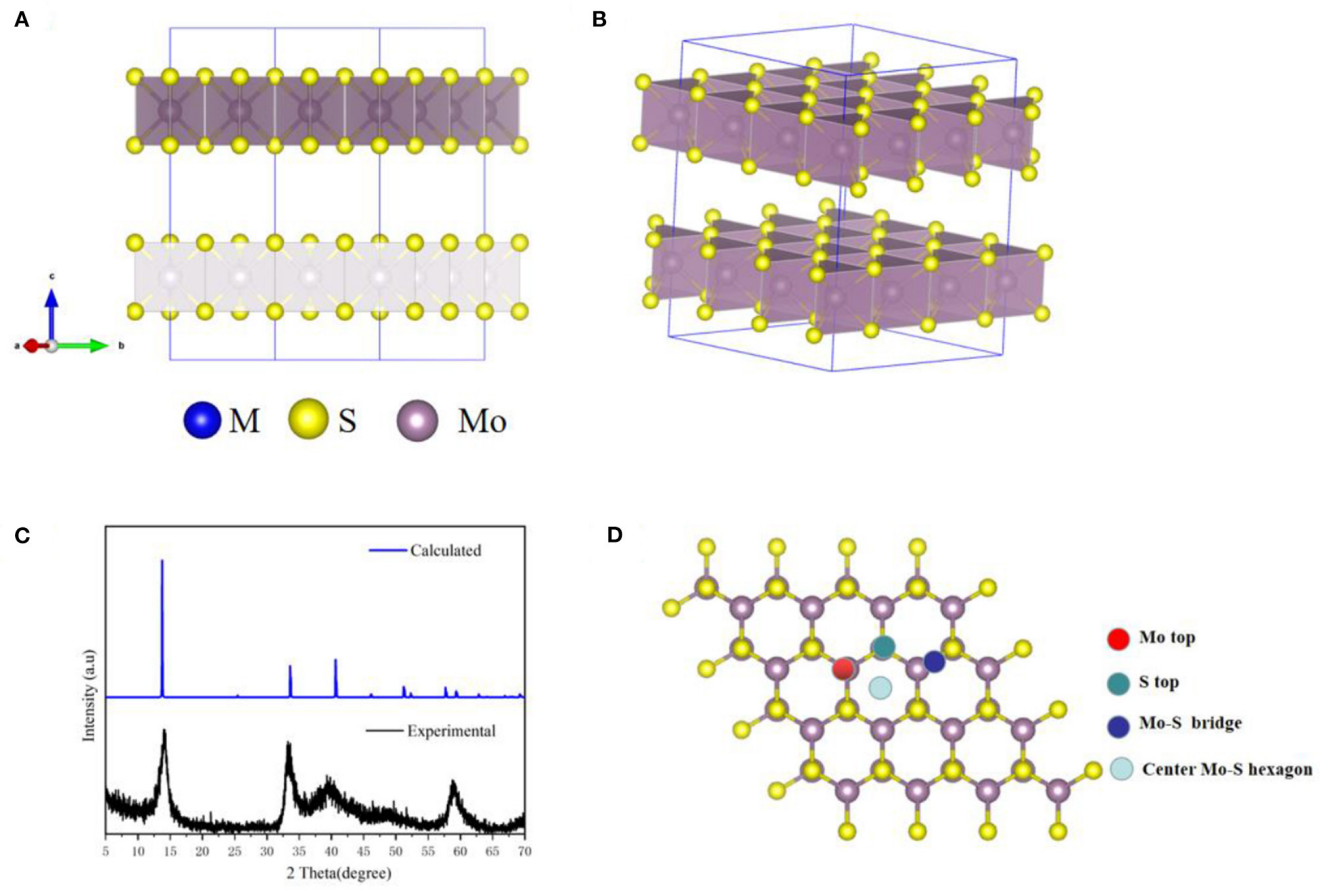
The MoS_2_ structure drawings viewed along **(A)** c-axis and **(B)** the stereo perspective. **(C)** The XRD pattern of the MoS_2_ sample. **(D)** Adsorption site of M-ions.

Our structures calculated have been fully relaxed using first-principles calculation. Figure 1 shows the optimized crystal structure of MoS_2_ (supercell), and the optimized lattice constants are a = b = 3.18 Å and c = 15.12 Å for unit-cells. In addition, the theoretical XRD pattern was calculated based on DFT, which was consistent with the experimental results shown in Figure 1C. In Figure 1D, it can be seen that there are four possible sites in the MoS_2_ crystal structure. The large adsorption energy of ions in anode plays a fundamental role in providing a high ion storage energy in M-ions batteries, and the determination of suitable adsorption sites is a premise for the first-principles prediction of adsorption energies based on DFT. Therefore, the adsorption of M ions (Li, Na, Mg, and Zn) on MoS_2_ was investigated in our work.

**Figure 2:**
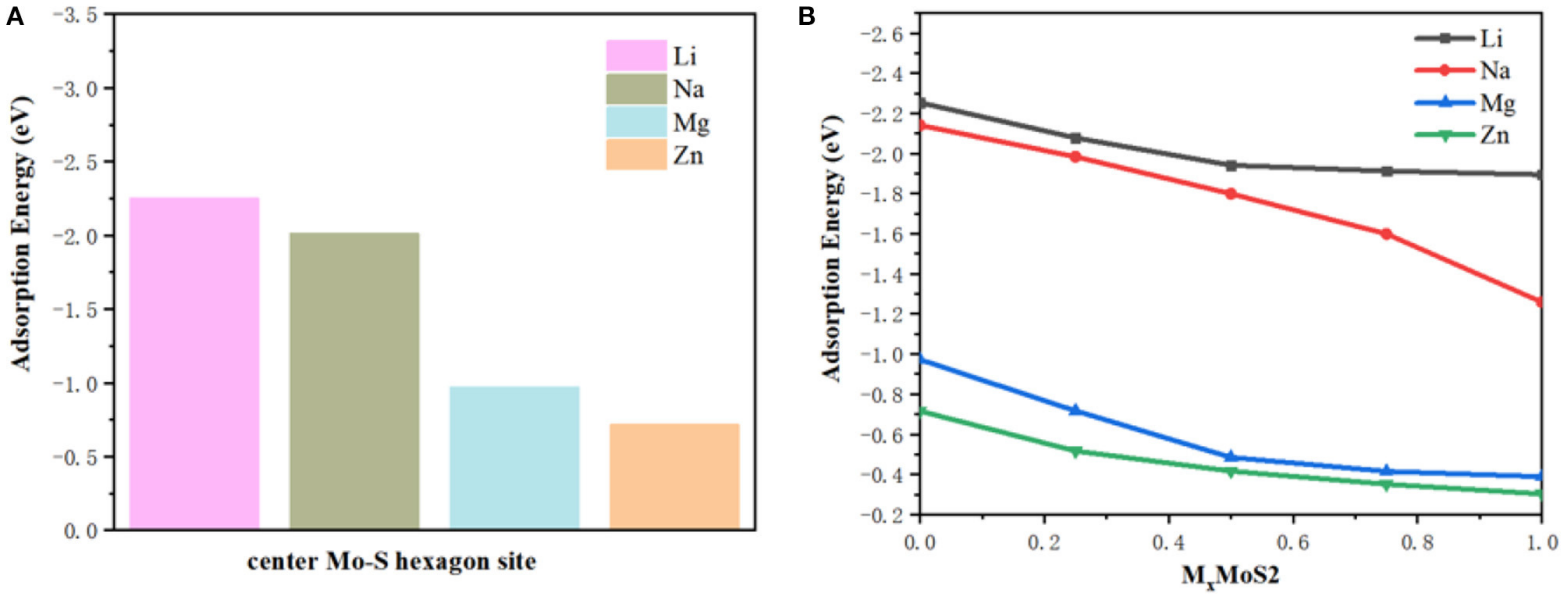
**(A)** The adsorption energies center Mo-S hexagon sites and **(B)** the adsorption energies with M-ions concentration.

The greater the negative adsorption energy, the better the thermodynamics and the more favorable for the adsorption thermodynamically. In our calculation, the 3 × 3 × 1 supercell for MoS_2_ was used to characterize the adsorption energy, corresponding to M_x_MoS_2_. Herein, as shown in Figure 1D, the different adsorption sites of metal on the MoS_2_ had been considered, including top site where metal atom sits directly above Mo (Mo top), hollow site above the center of Mo-S hexagon (center Mo-S hexagon), bridge site at the middle of Mo-S bond (Mo-S bridge) and the site directly above S atom (S top). And the adsorption energy had been shown in Table 1. Furthermore, the adsorption energy of Li and Na ions was found to be lower than that of Mg and Zn ions, implying the storage of Li and Na ions in the MoS_2_ anode material, thus resulting in higher specific capacity for Li- and Na- ions batteries in Figure 2A. In Figure 2B, the adsorption energies of M ions (Li, Na, Mg and Zn) were seen to increase gradually with the increase of M-ions concentration, and the large coulomb repulsion became increasingly apparent in the adjacent positively charged M-ions due to the high metal adsorption concentration. It noted that the Na curve decreases the fastest among others with higher adsorption concentration. And it may caused by the strong coulomb repulsion between Na neighboring positively charged, the large Na ion radius and adsorption energy. In addition, the adsorption energy of M-ions was lower than the cohesive energy of metal, such as Li metals (−2.01 eV), in a real battery system to ensure a positive discharge potential. When the number of atom adsorption increases, the adsorption energy of M-ions was larger than the cohesive energy of metal, which may form clusters. In Figure 2B, the adsorption energy of Zn-ions was shown to be larger than that of the other ions, suggesting that a small amount of Zn-ions can be stored in the MoS_2_ structure, leading to the low voltage and capacity of Zn-ions batteries. As a typical Li-ions batteries, we predict the capacity of MoS_2_ for Li batteries theoretically. From the Figure 2B, it is found that one Li atom adsorbed on a unit cell of MoS_2_, corresponding to storage capacity of ~687 mAh g^−1^, the adsorption energy becomes smaller than the chemical potential.

**Table 1:** The adsorption energies (eV) with different sites in MoS_2_ structure.

**Sites**	**Li**	**Na**	**Mg**	**Zn**
Mo top	−1.24	−0.61	−0.31	−0.28
S top	−1.08	−0.29	−0.17	−0.12
Mo-S bridge	−1.16	−0.55	−0.28	−0.26
Center Mo-S hexagon	−2.54	−2.02	−0.97	−0.71

**Figure 3:**
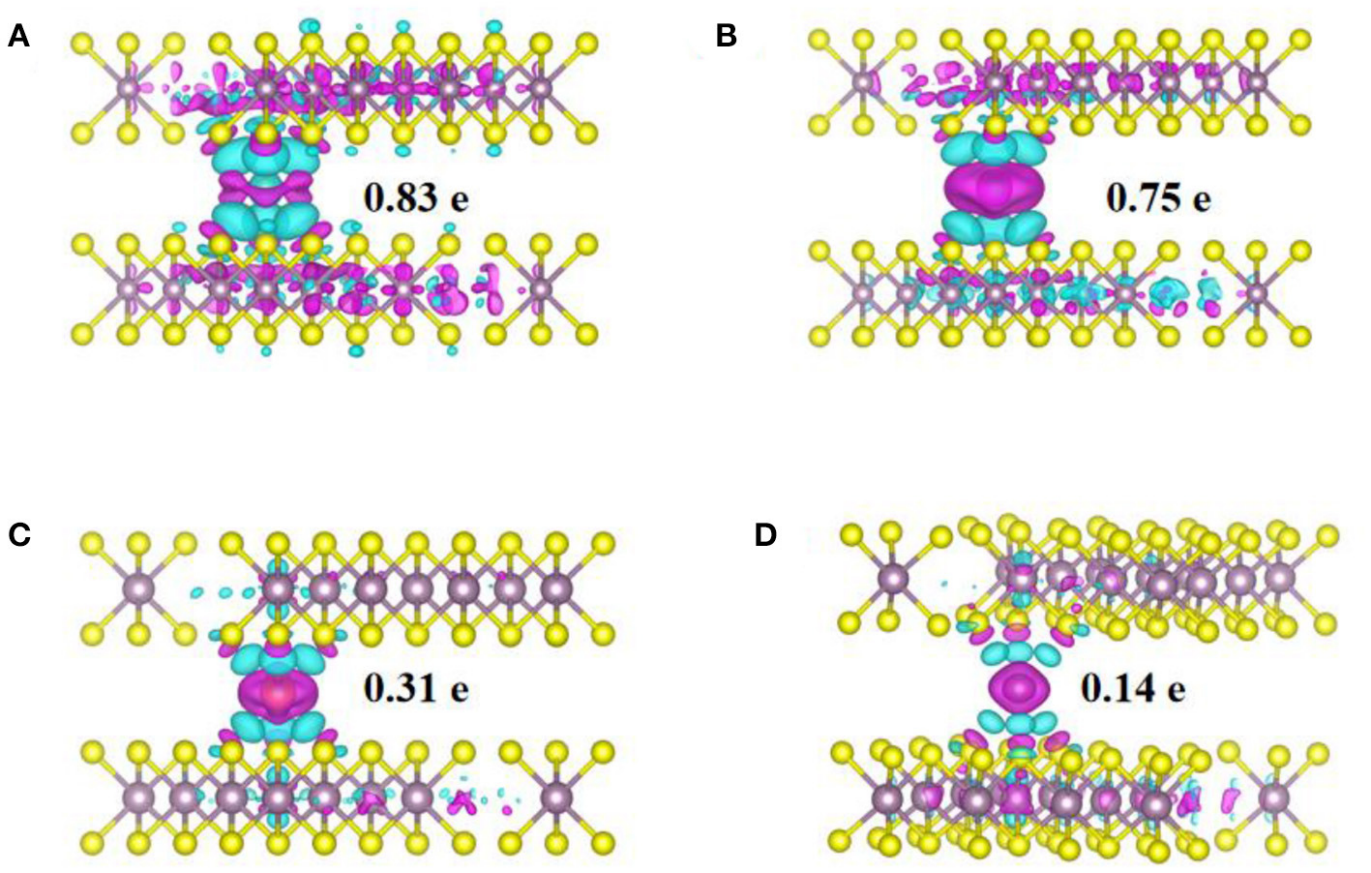
The charge density difference and bader charge transfer of M-ions in MoS_2_ structure. **(A)** Li-ion, **(B)** Na-ion, **(C)** Mg-ion, **(D)** Zn-ion.

**Figure 4:**
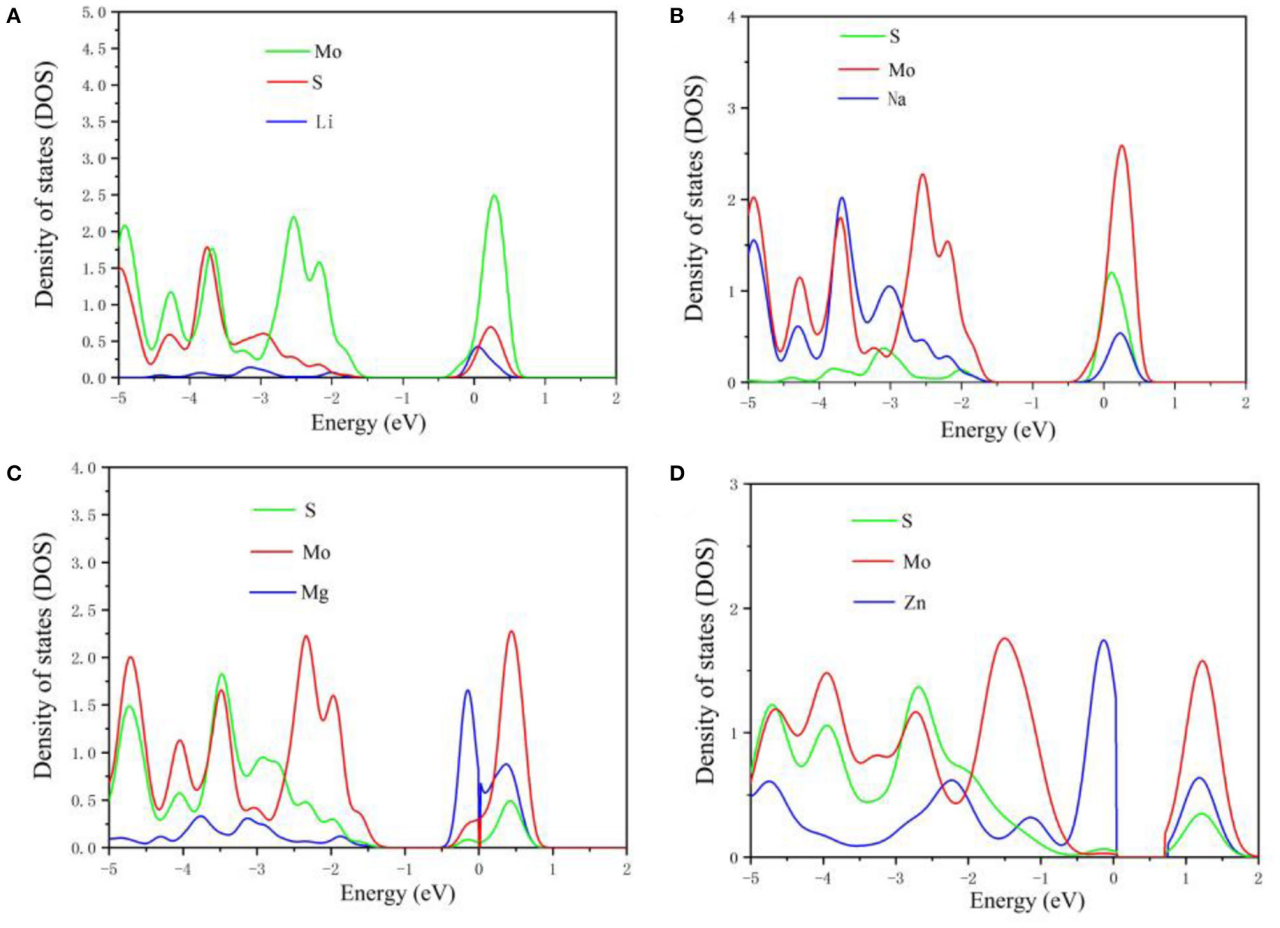
The projected density of states in MoS_2_ structure during Li **(A)**, Na **(B)**, Mg **(C)**, and Zn **(D)** intercalation.

To study the physical origin of ions adsorption and anode performance, the charge density differences and density of states (DOS) of the adsorption structures were calculated and shown in Figures 3, 4. Figure 3 shows the charge density differences obtained by subtracting the total electron densities of MoS_2_ and isolated metal (Li, Na, Mg, and Zn) atom in center of Mo-S hexagon sites from that of the M_x_MoS_2_ structure. The isovalue was set as 0.03 eÅ^−3^, charge depletion was in green and accumulation in red. the distribution was similar in the charge density difference between Li ions and Na ions. Additionally, the charge rehybridization during the intercalation of Mg and Zn ions was obviously greater than that of Li and Na ions. Furthermore, a considerable alteration can be observed in the charge accumulation region between Li/Mg ions and S atoms, because these atoms are closer to one layer of sulfurs in the MoS_2_ structure. However, in Figure 3B, the transferred electrons were shown to be largely localized for Na ions, thus reducing the energy consumption during Na-ions diffusion.

**Figure 5:**
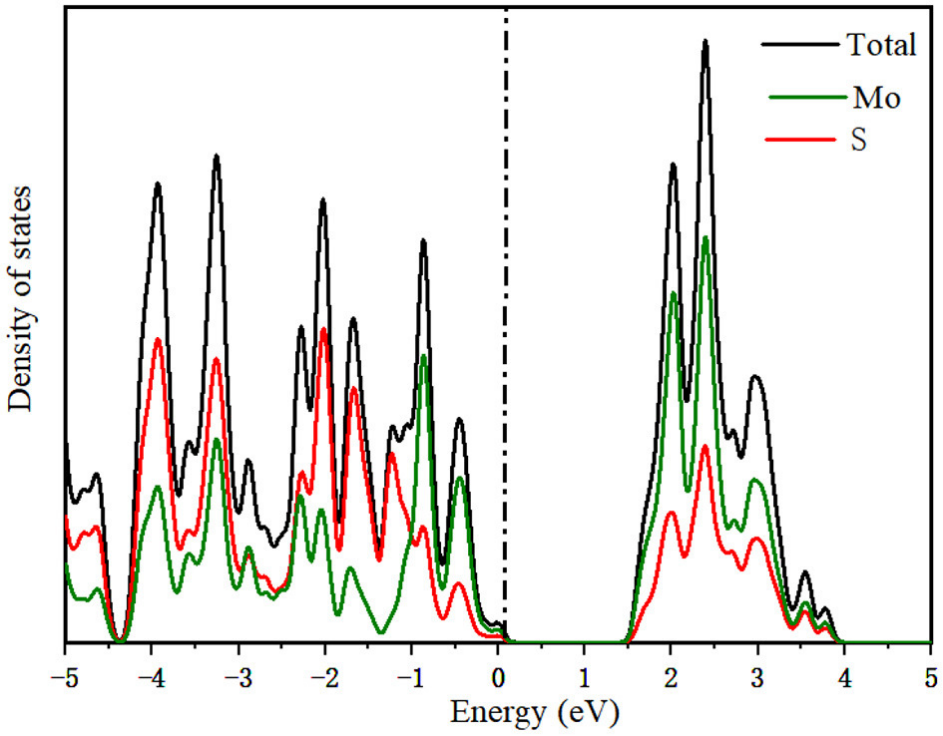
The projected density of states in MoS_2_ structure.

To further study the electronic structure of MoS_2_ during the intercalation of M-ions, the DOS was investigated (Figure 4), with the Fermi level set to zero. In Figures 4A,B, the band gap of MoS_2_ was seen to vanish during the adsorption of Li and Na-ions in contrast to the existence of the band gap in the adsorption of Mg and Zn-ions on MoS_2_. For comparison, the DOS of pure MoS_2_ had be repeated (Figure 5), which was in accordance with the reported previously (Hao et al., 2018; Chen et al., 2020). These results indicated that the semiconductor MoS_2_ may be transformed into metal during the insertion of Li and Na ions, while the properties of the MoS_2_ semiconductor remained unchanged during the insertion of Mg and Zn ions (Figure 4D), resulting in low electronic conductivity in divalent-ions batteries. Additionally, the conduction bands (CBM) were dominated by the Mo orbitals based on the DOS results. Meanwhile, the adsorbed ions (Li and Na) showed a discernible common peak between −3 and −1.5 eV with the S orbitals of MoS_2_, suggesting the hybridization between the adsorbed atoms and the MoS_2_ anode. However, the atoms adsorbed by Zn ions did not exhibit such characteristics. This is consistent with charge transfer analysis, where Li and Na loses electrons while MoS_2_ gains electrons with an itinerant feature. The electronic structures of MoS_2_ adsorbed by Li and Na ions were metallic, which can ensure a good electrical conduction, while the MoS_2_ adsorbed by Zn ions was shown to have semi-conductivity according to the electronic structure, leading to poor electrical conduction in Zn-ions batteries.

**Figure 6:**
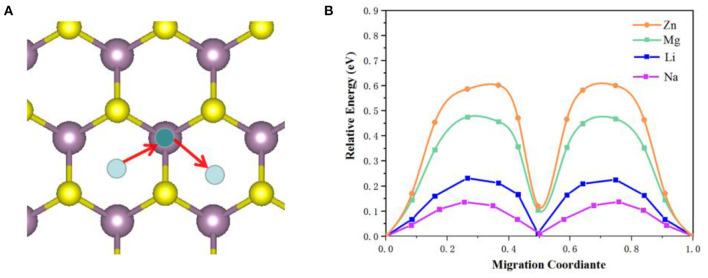
The M-ions migration pathways **(A)** and corresponding migration energy **(B)** in MoS_2_ structure.

For the MoS_2_ structure, the migration pathways of M-ions and the corresponding migration energy barriers were calculated using the CINEB method and shown in Figure 6. In Figure 6A the migration pathways of M-ions (Li, Na, Mg and Zn) were seen to move from the center Mo-S hexagon site to the adjacent Mo top site. Based on our DFT results, the migration energy barriers of M-ions were estimated and shown in Figure 6B, which were 0.22, 0.13, 0.47, and 0.61 eV for Li, Na, Mg and Zn ions, respectively. The energy barrier of Na ions was lower than that of the other ions and in the order of Zn>Mg>Li>Na, which was consistent with previous reports (Shu et al., 2016; Sun et al., 2018). It is worth noting that the migration barrier was lower than that of Li-ions in Na-ions, possibly due to the distribution of charge density differences. Besides, the divalent nature significantly induced charge rehybridization during the intercalation of Mg or Zn-ions, leading to sluggish mobility. These results agreed with the electronic structure analysis. Generally, the migration of ions inside the anode can directly describe the charging and discharging rates (C rate) for M-ions batteries, and thus the migration barrier of ions is always considered as a desirable design parameter.

**Figure 7:**
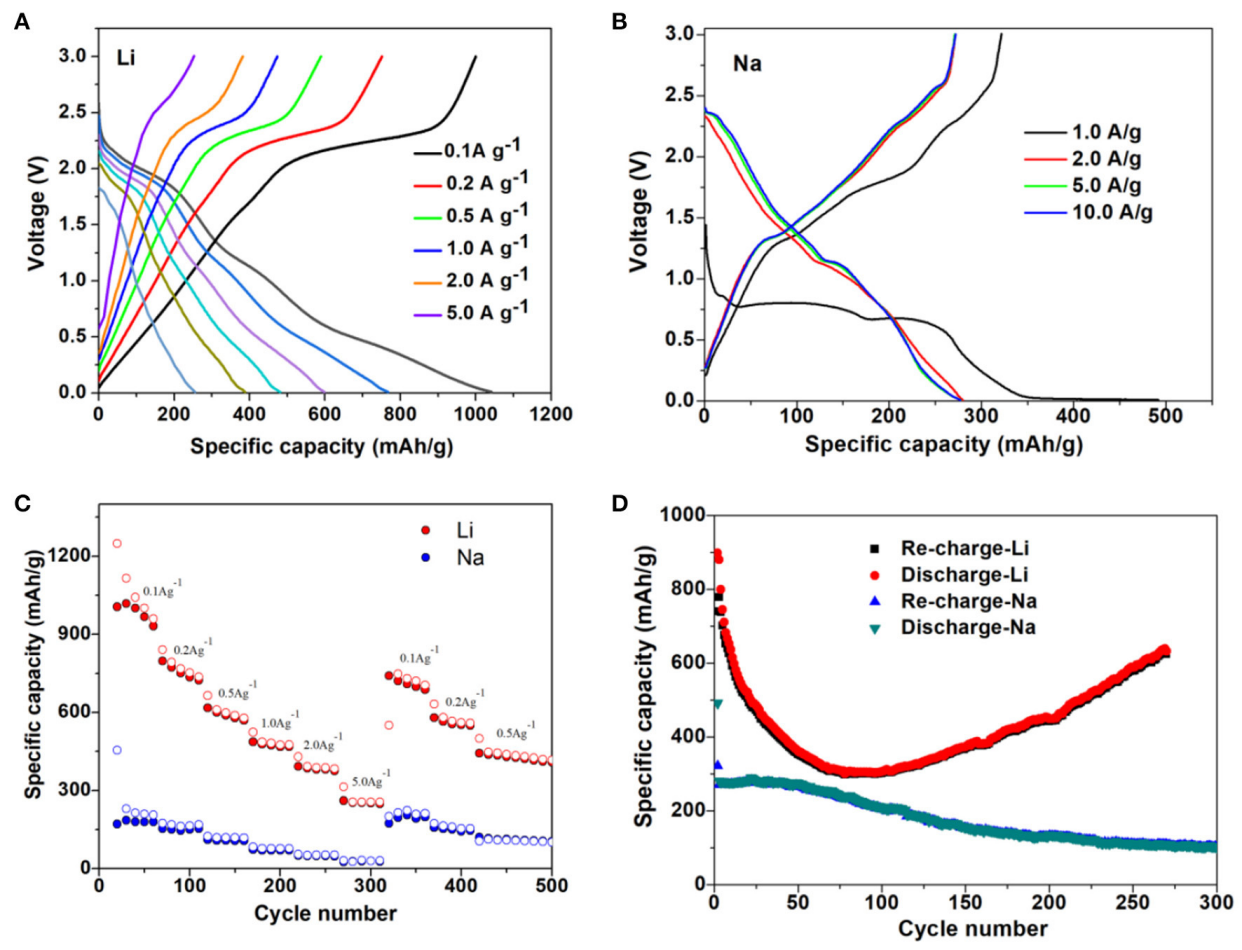
Galvanostatic charge-discharge curves of MoS_2_ at varied current density for **(A)** Li-ions battery anode and **(B)** Na-ions battery anode. **(C)** Rate properties of the MoS_2_ anode for Li- and Na-ions battery. **(D)** Cycling properties of MoS_2_ anode for Li- and Na-ions battery.

**Figure 8:**
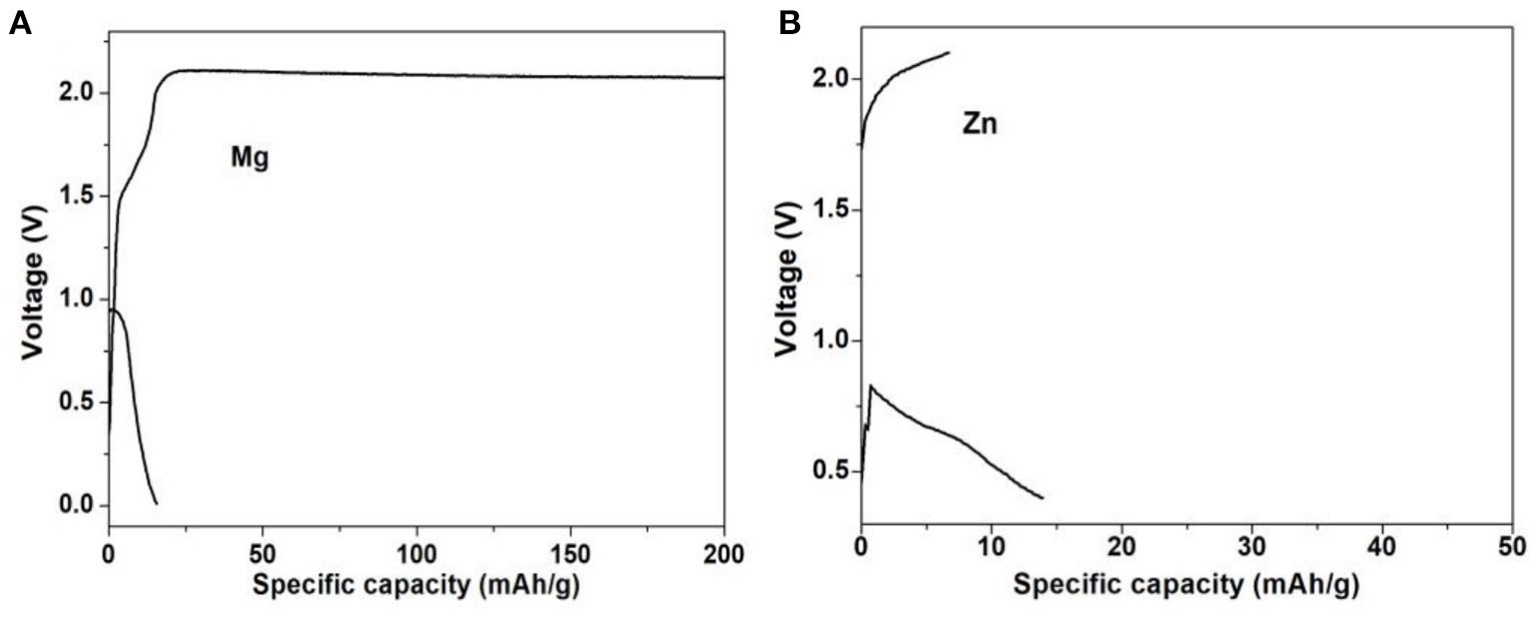
Galvanostatic charge-discharge curves of MoS_2_ for **(A)** Mg and **(B)** Zn-ions battery.

Furthermore, the electrochemical experiments were performed for M-ions batteries, and the electrochemical performances of Li- and Na-ions batteries with the MoS_2_ anode at different current densities were shown in Figures 7A–C. Even at the high current density of 2 A g^−1^ for Li-ions batteries and 10 A g^−1^ for Na-ions batteries, the discharge platform was still in good condition and could ensure effective ion/electron transmission. In Figure 7C, the capacity of the MoS_2_ electrode was seen to remain at a high value when the current density returned to 0.1 A g^−1^ for both Li- and Na-ions batteries, indicating the good rate capability. The long cycle performance of Li- and Na-ions batteries is shown in Figure 7D, with a good coincidence observed for the charge capacity and discharge capacity. In Figure 8, it can be seen that, compared with Li- and Na-ions batteries with MoS_2_, Mg- and Zn-ions batteries with the MoS_2_ anode, especially for the Zn-ions batteries, had very poor electrochemical performance. All these results were consistent with the theoretical calculation results.

### Structural Transformation

**Figure 9:**
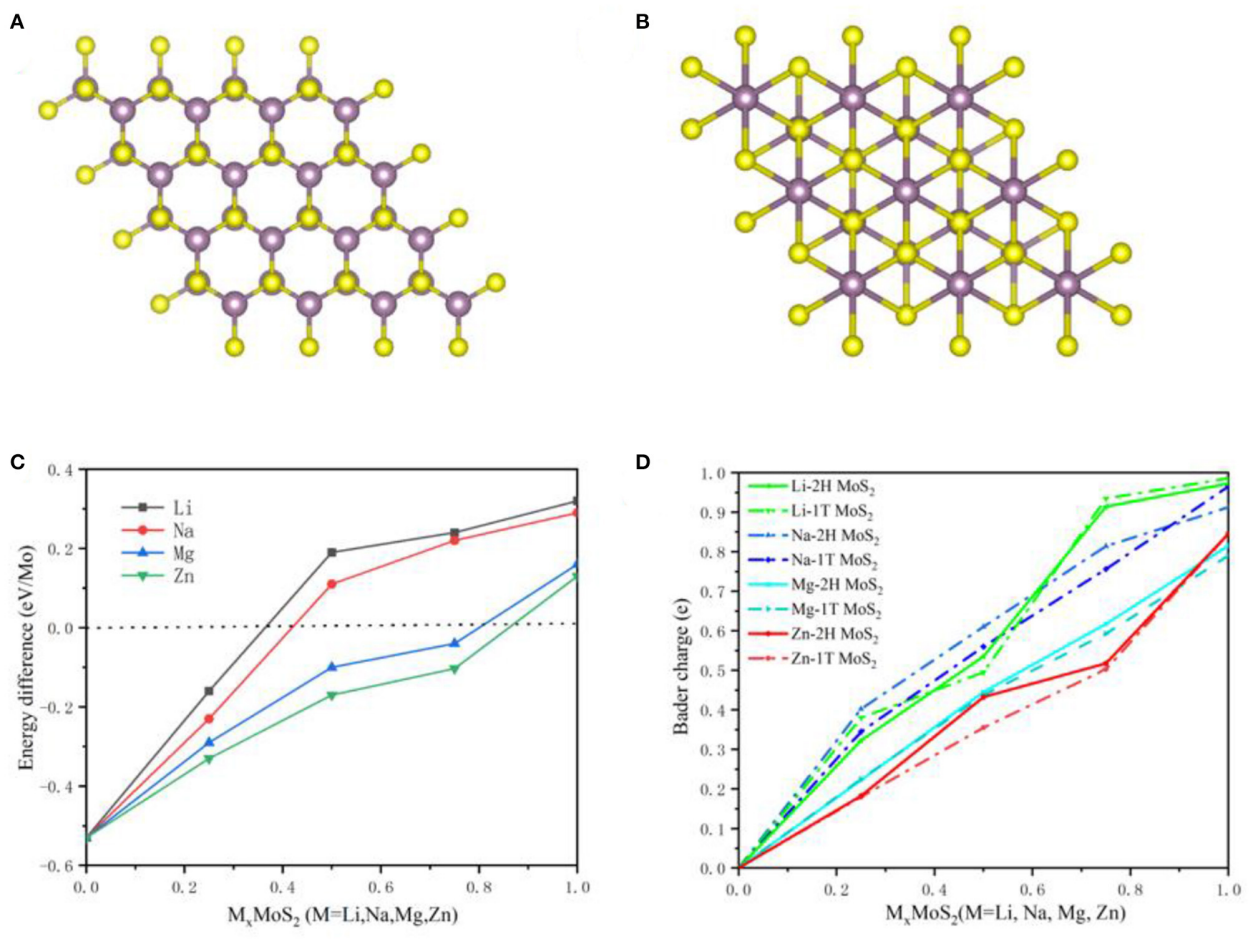
The 2H **(A)** and 1T **(B)** for MoS_2_ anodes structure. **(C)** Energy difference between the 2H and 1T structure of MoS_2_ with Metal ions concentration and **(D)** Charge transfer with M-ions concentration in 2H and 1T MoS_2_ structure.

Undoubtedly, the MoS_2_ anode underwent a phase transition from 2H to 1T during Li ions intercalation, which has been confirmed by theoretical calculations and experimental analysis (Wang et al., 2013; Du et al., 2016; Zhu et al., 2019). Figures 9A,B shows the 2H and 1T structures of MoS_2_, with a trigonal prism shape for 2H and an octahedron for 1T in the Mo coordination structure. In order to describe the phase transition in the MoS_2_ anode during M-ions intercalation, the energy difference between the 2H and 1T structures of MoS_2_ was calculated under different M-ions concentrations based on first-principles, and shown in Figure 9C. The energy of 2H was lower than that of the 1T structure at the early intercalation stage of M-ions, while the 1T structure obtained relatively more energy at a higher M-ions concentration, suggesting the occurrence of the transition from 2H to 1T. For Li-ions batteries, Li ions were adsorbed in the tetrahedral coordination center of the S-S, leading to the charge transfer from Li ions, which was confirmed by the above electronic structure analysis (Figures 3, 4). In addition, the Mo atom was found to be surrounded by six S atoms after lithiation, implying that the Mo coordination structure may be transformed from 2H to 1T due to the intercalation of Li ions and the transfer of electrons. The 1T-type MoS_2_ possessed a higher conductivity than the 2H-type MoS_2_ due to its disordered octahedral structure. Therefore, this conversion of MoS_2_ can improve the electronic conductivity. Moreover, the 1T structure of MoS_2_ was more stable in energy when intercalating Li ions. In Figure 9, the phase conversion of 2H to 1T was also shown to be faster in Na ions than in the other M-ions, corresponding to a relatively lower concentration of Na-ions, which was caused by the larger ionic radius and electron transfer in Na-ions batteries. Therefore, the total charge transfer from M-ions to MoS_2_ was calculated and shown in Figure 9D. However, in the case of a high concentration of Zn-ions and an abundant intercalation of Zn^2+^, the energy was still higher in 2H than in the 1T structure, indicating that it is difficult to convert 2H to 1T in the MoS_2_ structure. In Figure 9D, the Mo coordination structure showed no significant change during the intercalation of Zn-ions, with a small amount of charge transfer from Zn-ions to MoS_2_. The aforementioned results suggest that the phase conversion from 2H to 1T is beneficial to improve the performance of the MoS_2_ anode.

## Conclusion

In summary, the intercalation mechanism of metal ions (Li, Na, Mg, and Zn) and the intrinsic properties of MoS_2_ as an anode material in M-ions batteries were investigated by experiments and first-principles calculations, and the ground state properties, geometrical and electronic structures, as well as the intercalation mechanism of M-ions in MoS_2_ were explored by DFT. Based on the calculation of adsorption energies, the Li and Na ions were stored in the MoS_2_ anode material due to the low adsorption energies, which may result in higher specific capacity for Li- and Na-ions batteries, in contrast to a weak adsorption strength of MoS_2_ for Mg and Zn ions, which is not conducive to the storage of anode. These results were also confirmed by the electrochemical performance experiments. Moreover, the density of states and charge density differences were investigated to explore the intercalation mechanism of M-ions. Our results suggest the hybridization between the Li/Mg-ions and the MoS_2_ anode and a considerable alteration in the charge accumulation region due to the amount of charge transfer. However, such characteristics were not observed in the atoms adsorbed by Zn ions. Furthermore, the migration of M-ions was used to describe the charging and discharging rates (C rate) for M-ions batteries, and the energy barrier was shown to be in the order of Zn>Mg>Li>Na. Mg and Zn-ions had poor electrochemical performance due to weak adsorption and high energy barrier of ions, which also was confirmed by the electrochemical performance experiments. Interestingly, the phase conversion from 2H to 1T was found to occur during the intercalation of Li and Na ions, which may induce the high adsorption capacity and electron transfer, resulting in the high performance of the anode. However, the phase transformation failed to occur in Mg and Zn-ions batteries. In the future, we may improve the performance of multivalent-ions batteries by regulating the phase transformation of MoS_2_. For example, the intercalation of molecules between the layers can increase the interlayer spacing and facilitate the intercalation of ions and the transfer of electrons, thereby making it more prone to phase change. Overall, the intercalation mechanism of M-ions can help optimize the design of layered structure for the high performance of M-ion batteries, and shed light on more efficient battery technologies.

## Data Availability Statement

The original contributions generated for the study are included in the article/supplementary material, further inquiries can be directed to the Corresponding author.

## Author Contributions

BBC developed the idea for the study. JBZ did the analyses and wrote the paper. XDL, JJZ, HL, BWH, JQZ, and SMJ conceived of the study, designed the study, and collected the data. All authors analyzed the data and were involved in writing the manuscript.

## Conflict of Interest

The authors declare that the research was conducted in the absence of any commercial or financial relationships that could be construed as a potential conflict of interest.

## References

[B1] Chen J. Cao J. Zhou J. Wang W. Zhang Y. Liu X. (2020). Computational screening for enhanced hydrogen sensing by doped-2H and pristine,-1T., MoS. Chem. Phys. Lett. 16:137450. 10.1016/j.cplett.2020.137450

[B2] Cui C. Y. Wei Z. X. Xu J. T. Zhang Y. Q. Liu S. H. Liu H. K.. (2018). Three-dimensional carbon frameworks enabling MoS2 as anode for dual ion batteries with superior sodium storage properties. Energy Storage Mater. 15, 22–30. 10.1016/j.ensm.2018.03.011

[B3] Du H. Guo H. L. Liu Y. N. Xie X. Liang K. Zhou X.. (2016). Metallic 1T-LixMoS2 cocatalyst significantly enhanced the photocatalytic H-2 evolution over Cd0.5Zn0.5S nanocrystals under visible light irradiation. ACS Appl. Mater. Interfaces 8, 4023–4030. 10.1021/acsami.5b11377 26844371

[B4] Filippi C. Gonze X. Umrigar C. J. (1996). Generalized gradient approximations to density functional theory: comparison with exact results, in Theoretical and Computational Chemistry, ed Seminario J. M. (Amsterdam: Elsevier), 295–326. 10.1016/S1380-7323(96)80090-2

[B5] Forsyth M. Porcarelli L. Wang X. E. Goujon N. Mecerreyes D. (2019). Innovative electrolytes based on ionic liquids and polymers for next-generation solid -state batteries. Acc. Chem. Res. 52, 686–694. 10.1021/acs.accounts.8b00566 30801170

[B6] Grimme S. Ehrlich S. Goerigk L. (2011). Effect of the damping function in dispersion corrected density functional theory. J. Comput. Chem. 32, 1456–1465. 10.1002/jcc.21759 21370243

[B7] Hao J. Zheng J. Ling F. Chen Y. Jing H. Zhou T.. (2018). Strain-engineered two-dimensional MoS_2_ as anode material for performance enhancement of Li/Na-ion batteries. Sci. Rep. 8:2079. 10.1038/s41598-018-20334-z 29391534 PMC5794781

[B8] Henkelman G. Uberuaga B. P. Jonsson H. (2000). A climbing image nudged elastic band method for finding saddle points and minimum energy paths. J. Chem. Phys. 113, 9901–9904. 10.1063/1.1329672

[B9] Hu J. Liu Y. Liu N. Lia J. Ouyang C. (2020a). Theoretical prediction of T-graphene as a promising alkali-ion battery anode offering ultrahigh capacity. Phys. Chem. Chem. Phys. 22, 3281–3289. 10.1039/C9CP06099E 31970357

[B10] Hu J. Ouyang C. Yang S. A. Yang H. Y. (2019). Germagraphene as a promising anode material for lithium-ion batteries predicted from first-principles calculations. Nanoscale Horiz. 4, 457–463. 10.1039/C8NH00333E 32254098

[B11] Hu J. Zhong C. Wu W. Liu N. Liu Y. Yang S. A. (2020b). 2D honeycomb borophene oxide: a promising anode material offering super high capacity for Li/Na-ion batteries. J. Phys. 32:065001. 10.1088/1361-648X/ab4f4d 31631885

[B12] Hu L. R. Ren Y. M. Yang H. X. Xu Q. (2014). Fabrication of 3D Hierarchical MoS2/Polyaniline and MoS2/C architectures for lithium-ion battery applications. ACS Appl. Mater. Interfaces 6, 14644–14652. 10.1021/am503995s 25100439

[B13] Ju J. Ma J. Wang Y. Cui Y. Han P. Cui G. (2019). Solid-state energy storage devices based on two-dimensional nano-materials. Energy Storage Mater. 20, 269–290 10.1016/j.ensm.2018.11.025

[B14] Kresse G. Furthmüller J. (1996). Efficiency of ab-initio total energy calculations for metals and semiconductors using a plane-wave basis set. Comput. Mater. Sci. 6, 15–50. 10.1016/0927-0256(96)00008-0 9984901

[B15] Li X. Y. Peng K. (2018). Hydrothermal synthesis of MoS2 nanosheet/palygorskite nanofiber hybrid nanostructures for enhanced catalytic activity. Appl. Clay Sci. 162, 175–181. 10.1016/j.clay.2018.06.015

[B16] Manoj M. Jasna M. Anilkumar K. M. Abhilash A. Jinisha B. Pradeep V. S.. (2018). Sulfur-polyaniline coated mesoporous carbon composite in combination with carbon nanotubes interlayer as a superior cathode assembly for high capacity lithium-sulfur cells. Appl. Surf. Sci. 458, 751–761. 10.1016/j.apsusc.2018.07.113

[B17] Mohanapriya K. Jha N. (2019). Hierarchically hybrid nanostructure of carbon nanoparticles decorated graphene sheets as an efficient electrode material for supercapacitors, aqueous Al-ion battery and capacitive deionization. Electrochim. Acta 324:134870. 10.1016/j.electacta.2019.134870

[B18] Perdew J. P. Yue W. (1986). Accurate and simple density functional for the electronic exchange energy - generalized gradient approximation. Phys. Rev. B 33, 8800–8802. 10.1103/PhysRevB.33.8800 9938293

[B19] Schmuch R. Wagner R. Horpel G. Placke T. Winter M. (2018). Performance and cost of materials for lithium-based rechargeable automotive batteries. Nat. Energy 3, 267–278. 10.1038/s41560-018-0107-2

[B20] Shu H. B. Li F. Hu C. L. Liang P. Cao D. Chen X. S. (2016). The capacity fading mechanism and improvement of cycling stability in MoS2-based anode materials for lithium-ion batteries. Nanoscale 8, 2918–2926. 10.1039/C5NR07909H 26780964

[B21] Sun D. Ye D. L. Liu P. Tang Y. G. Guo J. Wang L. Z.. (2018). MoS2/graphene nanosheets from commercial bulky MoS2 and graphite as anode materials for high rate sodium-ion batteries. Adv. Energy Mater. 8:1702383. 10.1002/aenm.201702383

[B22] Sun Y. Guo S. H. Zhou H. S. (2019). Exploration of advanced electrode materials for rechargeable sodium-ion batteries. Adv. Energy Mater. 9:1800212. 10.1002/aenm.201800212

[B23] Tian B. Du W. Chen L. Guo J. Shu H. Wang Y.. (2020). Probing pristine and defective NiB6 monolayer as promising anode materials for Li/Na/K ion batteries. Appl. Surf. Sci. 527:146580. 10.1016/j.apsusc.2020.146580

[B24] Wang B. B. Zhang Y. Zhang J. Xia R. Y. Chu Y. L. Zhou J. C.. (2017). Facile synthesis of a MoS2 and functionalized graphene heterostructure for enhanced lithium-storage performance. ACS Appl. Mater. Interfaces 9, 12907–12913. 10.1021/acsami.7b00248 28375001

[B25] Wang H. T. Lu Z. Y. Xu S. C. Kong D. S. Cha J. J. Zheng G. Y.. (2013). Electrochemical tuning of vertically aligned MoS2 nanofilms and its application in improving hydrogen evolution reaction. Proc. Natl. Acad. Sci. U. S. A. 110, 19701–19706. 10.1073/pnas.1316792110 24248362 PMC3856830

[B26] Wang S. W. Yang B. C. Chen H. Y. Ruckenstein E. (2018). Popgraphene: a new 2D planar carbon allotrope composed of 5-8-5 carbon rings for high-performance lithium-ion battery anodes from bottom-up programming. J. Mater. Chem. A 6, 6815–6821. 10.1039/C8TA00438B

[B27] Wang Y. Zheng Y. Zhao J. Li Y. (2020). Assembling free-standing and aligned tungstate/MXene fiber for flexible lithium and sodium-ion batteries with efficient pseudocapacitive energy storage. Energy Storage Mater. 33, 82–87. 10.1016/j.ensm.2020.06.018

[B28] Xie X. Q. Ao Z. M. Su D. W. Zhang J. Q. Wang G. X. (2015). MoS2/graphene composite anodes with enhanced performance for sodium-ion batteries: the role of the two-dimensional heterointerface. Adv. Funct. Mater. 25, 1393–1403. 10.1002/adfm.201404078

[B29] Yao S. S. Cui J. Lu Z. H. Xu Z. L. Qin L. Huang J. Q.. (2017). Unveiling the unique phase transformation behavior and sodiation kinetics of 1D van der Waals Sb2S3 anodes for sodium ion batteries. Adv. Energy Mater. 7:1602149. 10.1002/aenm.201602149

[B30] Zhu X. J. Li D. Liang X. G. Lu W. D. (2019). Ionic modulation and ionic coupling effects in MoS2 devices for neuromorphic computing. Nat. Mater. 18:141. 10.1038/s41563-018-0248-5 30559410

